# Segregation of Virulent Influenza A(H1N1) Variants in the Lower Respiratory Tract of Critically Ill Patients during the 2010–2011 Seasonal Epidemic

**DOI:** 10.1371/journal.pone.0028332

**Published:** 2011-12-14

**Authors:** Antonio Piralla, Elena Pariani, Francesca Rovida, Giulia Campanini, Alba Muzzi, Vincenzo Emmi, Giorgio A. Iotti, Antonio Pesenti, Pier Giulio Conaldi, Alessandro Zanetti, Fausto Baldanti, and the Severe Influenza A Task Force

**Affiliations:** 1 Struttura Semplice Virologia Molecolare, Struttura Complessa Virologia e Microbiologia, Fondazione Istituto Ricovero e Cura a Carattere Scientifico Policlinico San Matteo, Pavia, Italy; 2 Dipartimento di Sanità Pubblica-Microbiologia-Virologia, Università degli Studi di Milano, Milan, Italy; 3 Struttura Complessa Direzione Medica di Presidio, Fondazione Istituto Ricovero e Cura a Carattere Scientifico Policlinico San Matteo, Pavia, Italy; 4 Struttura Complessa Anestesia e Rianimazione I, Fondazione Istituto Ricovero e Cura a Carattere Scientifico Policlinico San Matteo, Pavia, Italy; 5 Struttura Complessa Anestesia e Rianimazione II, Fondazione Istituto Ricovero e Cura a Carattere Scientifico Policlinico San Matteo, Pavia, Italy; 6 Struttura Complessa Anestesia e Rianimazione, Azienda Ospedaliera San Gerardo, Monza, Italy; 7 Laboratorio di Patologia Clinica, Microbiologia e Virologia Istituto Mediterraneo per i Trapianti e Terapie ad Alta Specializzazione, Palermo, Italy; University Hospital San Giovanni Battista di Torino, Italy

## Abstract

**Background:**

Since its appearance in 2009, the pandemic influenza A(H1N1) virus circulated worldwide causing several severe infections.

**Methods:**

Respiratory samples from patients with 2009 influenza A(H1N1) and acute respiratory distress attending 24 intensive care units (ICUs) as well as from patients with lower respiratory tract infections not requiring ICU admission and community upper respiratory tract infections in the Lombardy region (10 million inhabitants) of Italy during the 2010–2011 winter-spring season, were analyzed.

**Results:**

In patients with severe ILI, the viral load was higher in bronchoalveolar lavage (BAL) with respect to nasal swab (NS), (p<0.001) suggesting a higher virus replication in the lower respiratory tract. Four distinct virus clusters (referred to as cluster A to D) circulated simultaneously. Most (72.7%, n = 48) of the 66 patients infected with viruses belonging to cluster A had a severe (n = 26) or moderate ILI (n = 22). Amino acid mutations (V26I, I116M, A186T, D187Y, D222G/N, M257I, S263F, I286L/M, and N473D) were observed only in patients with severe ILI. D222G/N variants were detected exclusively in BAL samples.

**Conclusions:**

Multiple virus clusters co-circulated during the 2010–2011 winter-spring season. Severe or moderate ILI were associated with specific 2009 influenza A(H1N1) variants, which replicated preferentially in the lower respiratory tract.

## Introduction

The emergence of a new pandemic influenza A strain in 2009 has raised global concerns for health organizations. Although moderate in general, severe cases of influenza-like illness (ILI) during the 2009 pandemic have been associated with the emergence of specific virus variants. In particular, the D222G or N mutations have been observed in cases of severe disease [1]–[3]. Since its emergence, the 2009 influenza A(H1N1) virus has evolved and seven distinct clades have been reported so far [4], [5]. Early findings from the 2010–2011 season have shown the circulation of two virus variants in the UK as well as in New Zealand, Australia and Singapore [6], [7]. The reasons for this wide variation could be ascribed both to the global spread of 2009 influenza A(H1N1) virus and to its circulation in the human population for more than one year. The molecular characterization of circulating viral variants is crucial for a timely identification of any change that may increase virulence.

The aim of this study was to investigate the prevalence of 2009 influenza A(H1N1) variants with mutated hemagglutinin (HA) in upper and lower respiratory tract of patients with severe infections.

In the framework of the severe influenza A surveillance program in Lombardy (Northern Italy, nearly 10 million inhabitants out of a total of about 60 million), our reference laboratories collected respiratory samples from patients admitted to intensive care units (ICUs) as well as from inpatients hospitalized at units other than ICUs, and from patients with community acquired infections referred by sentinel practitioners. Patients were stratified according to strict clinical criteria, and 2009 A(H1N1) HA sequences were analyzed in severe cases and in a comparable number of moderate and mild cases.

Particular consideration was paid both to the three structural elements (190-helix, 220-loop and 130-loop) in the HA involved in receptor binding site (RBS) domains and to the four antigenic sites (Sa, Sb, Ca and Cb), where point mutations could alter the ability of the virus to infect cells and the antibody reactivity [8].

## Materials and Methods

### Study population and clinical outcome

From November 1, 2010 to March 6, 2011, nasal swab (NS) and/or bronchoalveolar lavage (BAL) specimens collected from 45 patients with severe ILI admitted to 24 ICUs in the Lombardy region were analyzed. A comparable number of patients with moderate ILI (N = 40) or mild ILI (N = 54) were analyzed in parallel. Analyses were carried out at S.S. Virologia Molecolare, Fondazione IRCCS Policlinico San Matteo, Pavia or Dipartimento di Sanità Pubblica-Microbiologia-Virologia, Università degli Studi di Milano.

Patients were stratified using the criteria proposed by Zarychanski et al. [9] as follows: i) severe ILI, consisting of critically ill patients with acute respiratory distress syndrome (ARDS) characterized by bilateral alveolar infiltrates on chest radiography and rapidly progressive hypoxemia [10], admitted to ICUs for mechanical ventilation or extra-corporeal membrane oxygenation (ECMO); ii) moderate ILI, (positive chest radiography and/or reduced O_2_ blood saturation, pO2<90%) consisting of lower respiratory tract infection requiring admission to units other than ICUs for antiviral and/or oxygen supplementation by non-invasive assisted ventilation procedures; and iii) mild ILI, consisting of upper respiratory tract infection reported by sentinel practitioners and not requiring admission to hospital and administration of antiviral drugs or oxygen supplementation.

### Ethics statement

The local Ethics Committee consent was not required since according to a Regional Surveillance and Preparedness Plan (DGR IX/1046, 22 Dec. 2010 and DGR 5988, 30 Jun 2011), diagnostic and clinical management of patients admitted at hospitals in the Lombardy Region with severe and moderate ILI included prospective influenza A detection, subtyping and sequencing which was centralized at the two Regional Reference Laboratories (Molecular Virology Unit, IRCCS Policlinico San Matteo, Pavia and Dipartimento di Sanità Pubblica-Microbiologia-Virologia, Università degli Studi di Milano, Milan). Mild respiratory infections were collected by sentinel pratictioners and anonymously analyzed at the reference laboratory in Milan, in the frame of the National Surveillance Plan (Influnet). Informed consent was not necessary since patients with severe and mild ILI were included in a Regional diagnostic and clinical management protocol. Data were anonymously analyzed according to a Regional Surveillance and Preparedness Plan. Mild ILI were collected and analyzed within the National Surveillance Plan (Influnet).

### Virus detection and quantification

Detection and quantification of influenza A viruses was routinely performed on NS specimens by a real-time RT-PCR developed at the Centers for Disease Control, Atlanta, USA and available on the WHO website: (http://www.who.int/csr/resources/publications/swineflu/CDCrealtimeRTPCRprotocol_20090428.pdf). In a subset of patients influenza A quantification could be performed on both NS and BAL specimens.

### Sequencing of the hemagglutinin gene

The 2009 influenza A(H1N1) HA gene was amplified directly from clinical specimens using the Superscript III One-step RT-PCR amplification kit (Invitrogen, Carlsbad, USA). Primer sequences were as follows: forward primer, HA-F0 (nt 1–28) 5′-ATGAAGGCAATACTAGTAGTTCTGCT-3′, reverse primer, HA-R0 (nt 1681–1657) 5′-GTAGAGACCCATTAGAGCACATCCA-3′. The PCR product was purified using a Microcon-100 microconcentrator following manufacturer's instructions (Millipore, Bedford, MA, USA). Purified PCR products were sequenced using the BigDye Terminator Cycle-Sequencing kit (Applied Biosystem, Foster City, USA) with an ABI Prism 3100 DNA sequencer (Applied Biosystems, Foster City, USA).

### Phylogenetic analysis

Sequences were assembled using the Sequencher software, version 4.6 (Gene Codes Corporation, Ann Arbor, USA). Nucleotide alignments were constructed using the ClustalW method with MEGA version 4.1 software [11]. Phylogenetic trees were generated using neighbour-joining distance method with a Kimura 2-parameter as an evolutionary model. The bootstrap analysis performed to evaluate the reliability of the inferred tree included 1,000 replicates. Amino acids were numbered starting after the signal peptide (DTLC). Potential N-linked glycosylation sites were predicted using the NetNGlyc 1.0 Server [12].

NCBI GenBank accession numbers for our nucleotide sequences are JF801855-JF801909 and JN017095-JN017181.

### Statistical analysis

Comparison of viral loads from paired respiratory samples was performed with the Wilcoxon rank sum test for continuous paired variables. The Fisher's exact test for categorical variables was used for analysis of mutation frequencies between groups of patients.

## Results

### Demographic, virologic and clinical characteristics of study patients

This study included 139 patients with a 2009 influenza A(H1N1) virus infection: 45 (32.4%) critically ill patients with severe ILI, 40 (28.8%) patients with moderate ILI and 54 (38.8%) patients with mild ILI.

As shown in Table 1, the median age of patients with severe ILI was significantly higher than that of patients with mild ILI, while no difference was observed in comparison with moderate ILI patients. The overall percentage of male patients (61.9%) was 1.6 fold greater than that of females (38.1%), and the male/female ratio was 1.8 in both severe and moderate ILI groups and 1.3 in the mild ILI group (Table 1). NS samples were available for all patients, as these specimens were routinely sent to the laboratories for diagnosis of 2009 influenza A(H1N1).

**Table 1:** Demographic and clinical data in groups of patients analyzed.

		Group of patients (no.)			P value^b^		
Data	All patients (N = 139)	Severe (45)	Moderate (40)	Mild (54)	Severe *vs* Mild	Severe *vs* Moderate	Moderate *vs* Mild
Gender Number (%)							
Male	86 (61.9%)	29 (64.4%)	26 (65.0%)	31 (57.4%)			
Female	53 (38.1%)	16 (35.6%)	14 (35.0%)	23 (42.6%)			
Age^a^							
Median (years)	47	52	49	34	**0.01**	ns	ns
Range	1 mo-85	25–83	1 mo-85	1–83			

mo, month.

a for one patient this information was not available.

b P value>0.10 is considered not significant (ns); significant P value under 0.05 is in bold.

**Figure 1:**
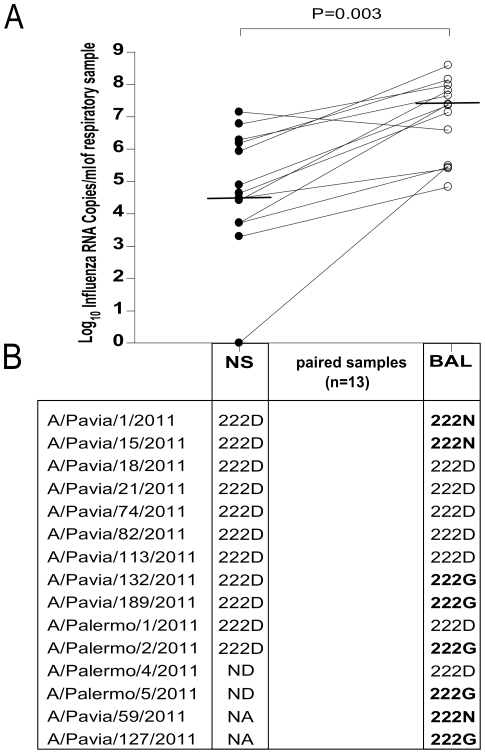
Paired samples analysis. A: Comparison of viral load in paired nasal swabs (NS) and bronchoalveolar lavage (BAL) samples. B: Distribution of 222 polymorphisms in paired NS and BAL specimens. NA, not available; ND, not done due to low viral load.

In the 13 patients with paired NS and BAL samples (12 severe ILI and 1 moderate ILI), virus load was significantly higher in the lower respiratory tract secretions (median 2.2×10^7^ RNA copies/ml, range 7×10^4^–3.8×10^8^ RNA copies/ml) than in upper respiratory tract samples (median 4.2×10^4^ RNA copies/ml, range 1–1.4×10^7^; p = 0.003) (Figure 1A).

### Phylogenetic analysis

The overall nucleotide identity of analyzed HA sequences with the reference vaccine strain A/California/07/2009(H1N1) ranged between 98.2% and 99.6%, with a maximum of 13 amino acid changes.

**Figure 2:**
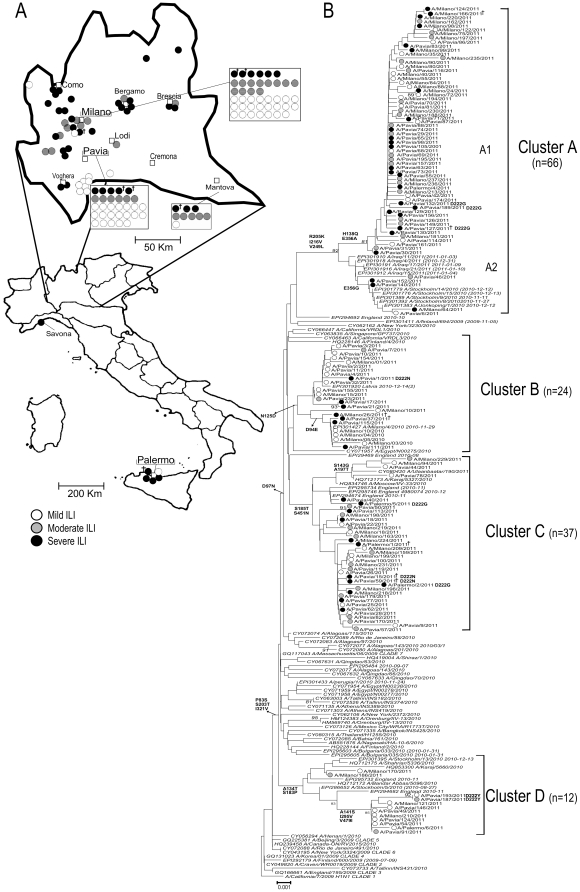
Geographic and phylogenetic strains distribution. A: Geographic distribution of patients categorized by clinical outcome. B: Phylogenetic tree based on influenza 2009 A(H1N1) viruses HA nucleotide sequences. Sequences of strains circulating in 2009–2011 are represented in italics (NCBI accession numbers or GISAID numbers are provided). NCBI GenBank accession numbers for our nucleotide sequences are JF801855-JF801909 and JN017095-JN017181. †, deceased patient.

As shown in figure 2A the patients categories were evenly distributed in the region and among the 24 ICUs. Phylogenetic analysis showed the simultaneous circulation of four distinct virus clusters referred to as clusters A, B, C and D (Figure 2B). Cluster A was characterized by the amino acid mutations R205K, I216V and V249L and included 66/139 (47.5%) sequences. Sequences belonging to cluster A were detected more frequently in patients with severe (n = 26) or moderate ILI (n = 22) than in those with mild ILI (n = 18) (72.7% vs 27.3%; p<0.001) (Figure 2B). Two sequences identified in deceased patients (A/Pavia/127/2011 and A/Milano/166/2011) fell into cluster A. Two sub-clusters (A1 and A2) were also described. Cluster A1 was characterized by H138Q and E356A substitutions, never reported previously. Cluster A2, characterized by mutation E356G, was closely related to a strain observed in Sweden (i.e. A/Stockholm/9/2010, A.N. EPI301389). Twenty-four (17.3%) virus sequences belonged to cluster B and were characterized by the N125D substitution (Figure 2B). In this cluster, two virus strains with an additional mutation (D94E) were observed in two deceased patients from different ICUs (A/Pavia/37/2011 and A/Milano/26/2011).

Cluster C, containing 37 (26.6%) sequences, was characterized by S185T and S451N substitutions, and corresponded to a cluster observed in the UK [6]. Three out of seven (42.9%) virus sequences from deceased patients belonged to this cluster. Two additional amino acid substitutions (S143G and A197T) were observed in three virus sequences from patients with mild ILI (A/Pavia/44/2011, A/Pavia/78/2011, and A/Milano/94/2011).

Cluster D, which included 12 (8.6%) sequences, was closely related to one of the two clusters circulating in the UK during the 2010–2011 seasonal epidemic [3]. This cluster was characterized by A134T and S183P mutations. Three additional mutations (A141S, I295V and V479I) were observed in 10/12 (83.3%) sequences. In contrast with the other three clusters, the substitution E374K was absent in this cluster. Nine out of 12 (75.0%) sequences in this cluster were from patients with mild ILI, while no sequences from patients with severe infections were detected in this cluster.

### Analysis of receptor binding site domains, antigenic epitopes and glycosylation sites

**Figure 3:**
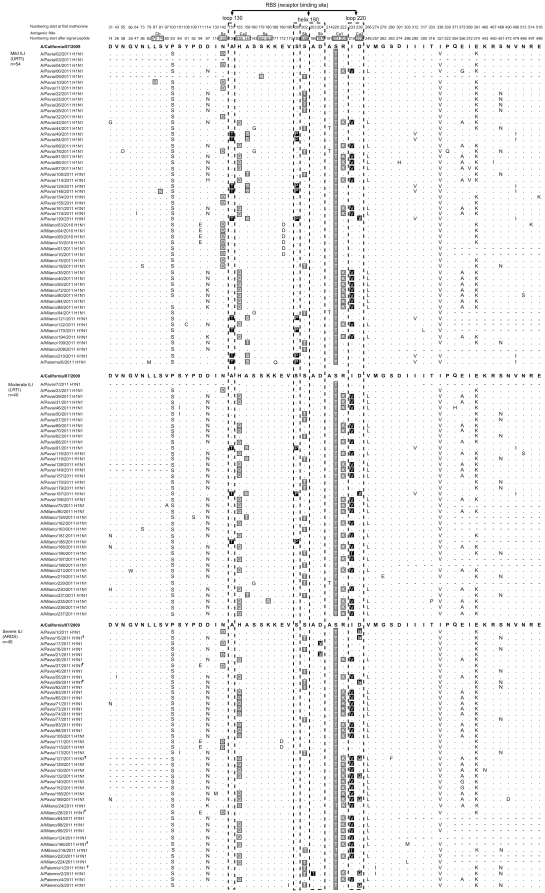
Amino acids changes with respect to vaccine strains (A/California/07/2009). The amino acid changes in the receptor binding sites (RBS) are highlighted in black. The amino acid changes in the antigenic sites are highlighted in grey. †, deceased patient; URTI, upper respiratory tract infection; LRTI, lower respiratory tract infection; ARDS, acute respiratory disease syndrome.

Most amino acid substitutions were located in the HA1 region containing the major antigenic epitopes. All sequences analyzed belonged to clade 7 and were characterized by the fixed amino acid mutation S203T (Figure 3).

Several HA mutations were observed throughout the three patient groups. On the whole, 59 HA residue positions were found with at least one change compared to vaccine reference strain A/California/07/2009(H1N1) (Figure 3). Overall, a greater number of changes was observed in patients with mild (41/59, 69.5%) than in patients with severe (28/59, 47.5%, p = 0.02) or with moderate ILI (32/59, 54.2%, p = 0.12).

The frequency of mutations in RBSs, antigenic sites and amino acids fixed in each cluster among patient groups was investigated (Table 2). The R205K, I216V, D222G, and V249L changes were found more frequently in patients with severe ILI than in those with mild ILI (p = 0.009). Conversely, the N125D, A134T, A141S/T, S183P, and I295V changes were detected more frequently in patients with mild ILI than in patients with severe ILI (p<0.05). The substitutions H138Q, R205K, I216V, D222G/N/Y, and V249L were identified more frequently in patients with severe and moderate ILI than in those with mild ILI (Table 2). Mutations V26I, I116M, A186T, D187Y, D222G/N, M257I, S263F, I286L/M, and N473D were observed only in patients with severe ILI (Figure 3). All these mutations except those at position 222 were present only in single or dual sequences.

**Table 2:** Prevalence of notable mutations in receptor binding sites, antigenic sites (Sa, Sb, Ca1, Ca2 and Cb) and amino acids fixed in each cluster among patient categories.

			Patient categories (no.)			P value^b^^,^ c			
Location of mutations		Mutations^a^	Severe (45)	Moderate (40)	Mild (54)	Severe *vs* Mild	Severe/Moderate *vs* Mild	Severe *vs* Moderate	Moderate *vs* Mild
Receptor Binding Sites	Loop 220	D222G/N/Y	8/45 (17.8%)	1/40 (2.5%)	1/54 (1.8%)	**0.01**	0.08	**0.03**	nns
		D222N	3/45 (6.7%)	0/40 (0%)	0/54 (0%)	0.09	ns	ns	ns
		D222G	5/45 (11.1%)	0/40 (0%)	0/54 (0%)	**0.02**	ns	0.06	ns
		D222Y	0/45 (0%)	1/40 (2.5%)	1/54 (1.8%)	ns	ns	ns	ns
		I216V	26/45 (57.8%)	22/40 (55.0%)	18/54 (33.3%)	**0.02**	**0.009**	ns	0.06
		I216T	1/45 (2.2)	1/40 (2.5%)	0/54 (0%)	ns	ns	ns	ns
	Helix 190	S183P	0/45 (0%)	3/40 (7.5%)	9/54 (16.7%)	**0.004**	**0.01**	0.10	ns
		A186T	1/45 (2.2%)	0/40 (0%)	0/54 (0%)	ns	ns	ns	ns
		D187Y	2/45 (4.4%)	0/40 (0%)	0/54 (0%)	ns	ns	ns	ns
	Loop 130	A134T	0/45 (0%)	3/40 (7.5%)	9/54 (16.7%)	**0.004**	**0.01**	0.10	ns
Antigenic sites	Sa	N125D	7/45 (15.5%)	1/40 (2.5%)	16/54 (29.6%)	**0.05**	**0.001**	0.06	**<0.001**
		S162N	0/45 (0%)	0/40 (0%)	1/54 (1.8%)	ns	ns	ns	ns
		K163R	0/45 (0%)	1/40 (2.5%)	0/54 (0%)	ns	ns	ns	ns
	Sb	S185T	12/45 (26.7%)	13/40 (32.5)	12/54 (22.2%)	ns	ns	ns	ns
		D187Y	2/45 (4.4%)	0/40 (0%)	0/54 (0%)	ns	ns	ns	ns
	Ca1	R205K	27/45 (60.0%)	22/40 (55.0%)	18/54 (33.3%)	**0.009**	**0.005**	ns	0.06
	Ca2	H138Q	21/45 (46.7%)	21/40 (52.5%)	17/54 (31.5%)	ns	**0.05**	ns	0.08
		A141S/T	0/45 (0%)	5/40 (12.5%)	10/54 (18.5%)	**0.001**	**0.02**	**0.02**	ns
	Cb	L70H	0/45 (0%)	0/40 (0%)	1/54 (1.8%)	ns	ns	ns	ns
		S74N	0/45 (0%)	0/40 (0%)	1/54 (1.8%)	ns	ns	ns	ns
	Cluster A	V249L	26/45 (57.8)	22/40 (55.0%)	18/54 (33.3%)	**0.03**	**0.009**	ns	0.06
		E356A/G^c^	20/38 (52.6%)	17/31 (54.8%)	17/45 (37.8%)	ns	ns	ns	ns
	Cluster B	D94E	4/45 (8.9%)	0/40 (0%)	4/54 (7.4%)	ns	ns	ns	ns
	Cluster C	S451N^c^	11/38 (28.9%)	9/32 (28.1%)	9/45 (20.0%)	ns	ns	ns	ns
	Cluster D	I295V^c^	0/43 (0%)	2/38 (5.3%)	8/52 (15.4%)	**0.007**	**0.01**	ns	ns

a Amino acids numbering start after signal peptide DTLC.

b P value>0.10 is considered not significant (ns); P value between 0.05 and 0.10 was considered a trend of significance; significant P value under 0.05 are in bold.

c Data not available for all sequences.

D222G/N amino acid changes were detected more frequently in sequences from patients with severe ILI (8/45; 17.7%) than in those from patients with mild (0/54, 0%; p = 0.01) or moderate ILI (0/40, 0%; p = 0.03). The D222G/N changes were observed only in BAL samples from five patients with severe ILI, while the HA sequences in the paired NS samples showed the wild-type 222D (Figure 1B). In addition, an amino acid change from aspartic acid (D) to tyrosine (Y) at residue 222 was observed in the HA sequences of viruses detected in two patients hospitalized in the same hematology ward at IRCCS Policlinico San Matteo: one presented with pneumonia and the other one with mild respiratory symptoms. Interestingly, the HA sequences carrying mutations at residue 222 did not segregate in a particular virus cluster, but were evenly distributed in clusters A, B and C (Figure 2B).

Besides D222G/N changes, an additional mutation in the RBS domain (D187Y) was observed. It was detected only in viruses from two patients with severe ILI, one requiring mechanical ventilation and the other requiring an ECMO procedure.

To address the difference in antigenic properties, the mutations in antigenic epitope regions (Sa, Sb, Ca1, Ca2 and Cb) were investigated (Figure 3). In all sequences, the change S203T in the antigenic site Ca1 was present as a fixed mutation characterizing sequences belonging to clade 7. A maximum of 10 mutations (excluding the S203T) were found in the antigenic sites. In particular, two in antigenic site Sa (N125D, and S162N ), two in antigenic site Sb (S185T and D187Y), one in antigenic site Ca1 (R205K), three in antigenic site Ca2 (H138Q, A141S/T, and D222G/N/Y), and two in antigenic site Cb (L70H and S74N). Three of these substitutions were found exclusively in patients with mild ILI (L70H, S74N, and S162N).

Six potential N-glycosylation sites were found in all HA sequences analyzed and corresponded with positions 11, 23, 87, 277, 287, and 482. The sequence named A/Pavia/09/2011 possessed an additional N-glycosylation site at residue 163 due to an amino acid change from serine (S) to asparagine (N), with a glycosylation score of 0.727. This patient presented with mild symptoms of ILI.

## Discussion

This study was aimed at investigate the prevalence of 2009 influenza A(H1N1) variants with mutated hemagglutinin (HA) in upper and lower respiratory tract of patients with severe infections in the 2010–2011 seasonal epidemic to extend observations reported during the 2009 pandemic [1]–[3].

In keeping with preliminary data published in the ECDC report released in February [13], the simultaneous circulation of four 2009 influenza A(H1N1) clusters in a geographical area including approximately 1/6 of the total Italian population (about 10/60 million inhabitants) was observed. Cluster A, characterized by mutations R205K, I216V and V249L, included about half of the analyzed HA sequences. Interestingly, nearly 70% of the patients infected with viruses belonging to this cluster developed either a severe or a moderate ILI. In contrast, clusters B, C, and D were observed with similar frequency in all patients categories. Cluster C represented about one-fourth of analyzed sequences. This result is partially in agreement with the ECDC report that described viruses harboring the S185T mutation as the variants showing the greatest expansion and geographic spread in Europe [13]. Of note, about half of deceased patients in both our series and the UK series were infected by viruses belonging to this cluster [6], [13]. Cluster D, characterized by A134T and S183P substitutions, was closely related to a cluster recently reported to circulate in the United Kingdom, Australia, New Zealand and Singapore [6], [7], [13]. The substitution E374K, previously reported to alter the stability of HA trimer [14] which was fixed in the others three clusters, was absent in sequences belonging to cluster D.

In the molecular analysis of HA sequences, particular attention was paid to the amino acid variations occurring in the HA structural elements involved in both the receptor-binding sites and the antigenic sites. Some of the substitutions observed were prominently exposed on the surface of the HA trimer (at residue 125, 134, 183, 185, 216) but others were buried. With respect to the reference vaccine strain A/California/07/2009(H1N1), a higher number of amino acid changes in the antigenic sites and, to a lower extent, in RBSs was observed in virus strains from patients with mild ILI than in those with severe or moderate ILI. This suggests that HA divergence in virus strains sustaining mild upper respiratory tract infections thus expressing a great spreading capacity might be the result of multiple rounds of selection by herd immunity. In contrast, the HA sequences of virus strains from patients with a severe infection were genetically more conserved, probably due to the evolutionary bottleneck represented by the receptor specificity. In fact, virus variants associated with severe infection are sequestered in the lower respiratory tract and, hence, the switch of receptor specificity of these viruses from α2-6 to α2-3-linked sialosides seems to decrease the capacity of the virus to infect the upper respiratory tract, thus impairing the spreading ability of those aggressive variants.

Consistent with data reported during the 2009 pandemic [1], [2], [15], the D222G/N variants were more frequently detected in patients with a severe influenza infection than in those with mild ILI. In addition, the analysis of paired NS and BAL samples showed that the 222G/N substitutions were found exclusively in the lower respiratory tract specimens supporting the hypothesized switch of receptor specificity from α2-6 to α2-3-linked sialosides [3].

In our series, D222Y variants, previously reported in severe cases [5], were detected in two epidemiologically but not clinically linked patients, since they attended the same hospital ward but had different clinical outcomes. None of these two patients developed ARDS and, thus, our data could not corroborate the increased virulence of these variants. Although the D187Y substitution was detected exclusively in two patients with severe ILI, no conclusive considerations on the increased virulence of these variants can be drawn. This amino acid substitution has been previously detected in a virus circulating in Brazil (A/Brasil/7450/2009) and, more recently, in an isolate from England (A/England/4500186/2010). However, no clinical data from patients carrying this viral variant were available.

Further studies on the receptor binding specificity and affinity will clarify both the role of such HA mutants on viral tropism and their contribution to exacerbation of disease.

In this study, patients with severe influenza infections showed a significantly higher viral load in lower respiratory tract secretions compared to paired upper respiratory tract ones. These findings confirm and extend the data found in the 2009 influenza pandemic [2], and further stress the importance of systematically analyzing BAL samples in patients with severe influenza A infection. It is worth mentioning that influenza surveillance data are based upon the analysis of upper respiratory tract samples only [6], [7] and thus, could fail to spot the emergence of more aggressive variants in the lower respiratory tract [16].

A limitation of this study is the relatively small set of data analyzed and the pathogenetic role of the virus mutants observed remains to be confirmed in a larger cohort. On the other hand, the infection by the 2009 influenza A(H1N1) strain was moderate in general and the prospective enrollment, according to strict clinical criteria, of 45 critically ill patients required the combined effort of 24 ICUs. Thus, this data set, although limited, appears unique to shed more light on the mechanisms of virulence of 2009 influenza A(H1N1). Although the comparison of different respiratory specimens might be questionable, our data are in agreement with results by Giannella et al [17] describing a reduced viral load in the upper vs lower respiratory tract of patients with severe influenza infections. Finally, others factors such as the host immune response and the existence of co-morbidities should be taken into account when evaluating severe infection outcome.

In conclusion, our results highlight that HA D222G/N mutation was detected exclusively in lower respiratory tract secretions. The findings of this study on the circulation of new variants of 2009 influenza A(H1N1) stress the importance of continuous monitoring of influenza virus evolution. Prompt analysis of severe infections by adopting appropriate sampling guidelines will promote better understanding of the epidemiology of mutated viruses with respect to clinical outcome.
